# Integrative Analysis Reveals Potentially Functional N6-Methylandenosine-Related Long Noncoding RNAs in Colon Adenocarcinoma

**DOI:** 10.3389/fgene.2021.739344

**Published:** 2021-09-17

**Authors:** Xinjie Tan, Qian Li, Qinya Zhang, Gang Fan, Zhuo Liu, Kunyan Zhou

**Affiliations:** ^1^ School of Medicine, Nankai University, Tianjin, China; ^2^ Department of Pediatrics, The Second Affiliated Hospital of Zheng Zhou University, Zhengzhou, China; ^3^ Department of Anesthesiology, Heidelberg University Hospital, Heidelberg, Germany; ^4^ Department of Anesthesiology, Affiliated Hospital of Guizhou Medical University, Guiyang, China; ^5^ The Affiliated Cancer Hospital of Xiangya School of Medicine, Central South University, Hunan Cancer Hospital, Changsha, China; ^6^ Department of Urology, Huazhong University of Science and Technology Union Shenzhen Hospital, The 6th Affiliated Hospital of Shenzhen University Health Science Center, Shenzhen, China; ^7^ Third Department of General Surgery, The Central Hospital of Xiangtan, Xiangtan, China

**Keywords:** N6-methylAdenosine (m6A), colon adenocarcinoma, long noncoding RNAs, metabolic reprogramming, m6A-related lncRNAs

## Abstract

N6-methyladenosine (m6A) is one of the most prevalent RNA modifications in mRNA and non-coding RNA. In this study, we identified 10 upregulated m6A regulators at both mRNA and protein levels, and 2,479 m6A-related lncRNAs. Moreover, the m6A-related long noncoding RNAs (lncRNAs) could clearly stratify the colon adenocarcinoma (COAD) samples into three subtypes. The subtype 2 had nearly 40% of samples with microsatellite instability (MSI), significantly higher than the two other subtypes. In accordance with this finding, the inflammatory response-related pathways were highly activated in this subtype. The subtype-3 had a shorter overall survival and a higher proportion of patients with advanced stage than subtypes 1 and 2 (*p*-value < 0.05). Pathway analysis suggested that the energy metabolism-related pathways might be aberrantly activated in subtype 3. In addition, we observed that most of the m6A readers and m6A-related lncRNAs were upregulated in subtype 3, suggesting that the m6A readers and the m6A-related lncRNAs might be associated with metabolic reprogramming and unfavorable outcome in COAD. Among the m6A-related lncRNAs in subtype 3, four were predicted as prognostically relevant. Functional inference suggested that CTD-3184A7.4, RP11-458F8.4, and RP11-108L7.15 were positively correlated with the energy metabolism-related pathways, further suggesting that these lncRNAs might be involved in energy metabolism-related pathways. In summary, we conducted a systematic data analysis to identify the key m6A regulators and m6A-related lncRNAs, and evaluated their clinical and functional importance in COAD, which may provide important evidences for further m6A-related researches.

## Introduction

Non-coding RNAs longer than 200 nucleotides are defined as lncRNAs, and they are transcribed from intergenic regions, or from any part of both sense or antisense DNA strand of protein coding genes (Panni et al., 2020). As an important member of the family of non-coding RNAs, lncRNAs are not translated into proteins, but they regulate gene expressions at both transcriptional and post-transcriptional levels (Dykes and Emanueli, 2017). Due to their diverse nature, functions of lncRNAs are different. They are capable of binding to proteins, directly targeting mRNA for degradation, harboring intronic miRNAs and possibly regulating gene expression through competing endogenous RNA (ceRNA) networks (Dykes and Emanueli, 2017). Of note, lncRNAs seem to play important roles in tumor progression and metastasis, as they are associated with critical cancer-related cell signaling pathways, including the p53, NF-κB, PI3K/AKT and Notch pathways (Peng et al., 2017).

N6-methyladenosine (m6A, methylation of the adenine base at the nitrogen 6 position) is one of the most prevalent chemical modifications on RNAs, especially on messenger RNAs (mRNAs) and long non-coding RNAs (lncRNAs) (Lence et al., 2019; Wen et al., 2020). During such modification, there are methyltransferase and demethylases complexes serving as “writers” and “erasers”, which install or remove m6A so as to dynamically regulate RNA modification levels (Yang et al., 2018a; Shi et al., 2019). Also, specific binding proteins are referred to as m6A “readers”, which recognize and bind their target RNA (Yang et al., 2018b; He et al., 2019; Liu et al., 2020). Recently, emerging evidence has suggested that m6A participates in cancer pathogenesis and progression. One example is the up-regulation of YTHDF1, a m6A reader, which could lead to unfavorable prognoses in ovarian cancer via augmenting the modification of EIF3C and facilitating tumorigenesis (Liu et al., 2020).

Associations between lncRNAs and the m6A modification in tumorigenesis has become a heated research field with advances in MeRIP-seq method. lncRNAs can be m6A-modified, which may directly alter their structures, stability, subcellular distribution, and affect their interaction with other molecules (He et al., 2020). It is found that m6A modification in GAS5, a tumor suppresser, would lead to GAS5 degradation via binding to m6A reader YTHDF3 in colorectal cancer (CRC), which further results in the decreased expression of YAP (Ni et al., 2019). YAP is known to promote the proliferation, invasion, and metastasis of COAD (Li et al., 2020). Meanwhile, dysregulated lncRNA expression would exert oncogenic effects via influencing m6A modification in certain mRNAs. According to a previous study, a 71-amino acid peptide encoded by lncRNA LINC00266-1 could interact with m6A reader IGF2BP1 to strengthen m6A recognition, which leads to increased expression of c-Myc and thus promotes tumorigenesis in colorectal cancer (Zhu et al., 2020). Moreover, m6A was also observed to promote the expression of RP11, which regulated Siah1-Fbxo45/Zeb1 stability in the development of CRC (Wu et al., 2019). In addition, m6A-related lncRNA signature could serve as novel biomarkers for predicting prognosis and immune response in COAD (Zhang et al., 2021). Therefore, it is urgent to systematically identify the key m6A regulators and m6A-related lncRNAs in COAD from the high-throughput data, explore the potential regulatory mechanisms that linked m6A regulators and lncRNAs, and to infer the roles of the m6A-related lncRNAs in the tumorigenesis and progression of COAD.

## Materials and Methods

### Data Collection

The gene expression data from the Cancer Genome Atlas (TCGA) (Cancer Genome Atlas Network, 2012) and the protein expression data from Clinical Proteomic Tumor Analysis Consortium (CPTAC) (Vasaikar et al., 2019) were downloaded from TCGA data portal (https://portal.gdc.cancer.gov/) and LinkedOmics (http://linkedomics.org), respectively. The expression levels of genes and proteins data were pre-normalized into the form of log2 (1 + Fragment Per Kilo-Million (FPKM)) and log2 intensity.

### Differential Expression Analysis

Prior to differential expression analysis, the genes with low abundance were excluded if their expression were below one FPKM in more than 85% of the total number of samples. The differential expression analysis was conducted using R limma method (Ritchie et al., 2015). The student t test and log2 fold change (FC) were applied to measure the differences. The genes with an adjusted *p*-value < 0.05 and a log2(FC) > 0.5 were considered as differentially expressed genes/proteins.

### Consensus Clustering

The consensus clustering algorithm was applied to determine the cancer subtypes using NMF method in R CancerSubtypes package (Xu et al., 2017). The optimal clustering number was determined according to the delta area plot.

### Prediction of the Interaction Between N6-Methyladenosine Proteins and Long Noncoding RNAs

The pairwise correlation between lncRNAs and m6A proteins were conducted using Spearman’s correlation analysis. Moreover, the physical interactions between the lncRNAs and m6A proteins were predicted by LncADeep (Yang et al., 2018b).

### Pathway Enrichment Analysis

Two pathway enrichment methods were used for the data analysis, as they used different statistical testing approaches to analyze different types of data. The first is overrepresentation enrichment analysis (ORA), which used a given gene set as input, and identified Reactome pathways enriched by this gene set, while such gene sets consisted of significantly upregulated genes across the subtypes. The second is the gene set enrichment analysis (GSEA), which used all genes sorted by the statistics of interest in descending order, such as the correlation coefficient, and identified a gene subset significantly enriched in the head or tail of the ordered genes, along with significantly enriched pathways in this gene subset. These two analyses were implemented in R clusterProfiler package (Yu et al., 2012). The pathway activities were estimated by single-sample enrichment analysis (ssGSEA), which were implemented in R GSVA package (Hänzelmann et al., 2013).

### Survival Analysis

The Cox proportional hazard regression model was employed in the survival analysis. The response variable is the survival time and status, and the predictive variables were the identified subtypes and expression levels of certain genes (high vs low, as compared to the median of gene expression). The survival analysis and visualization were implemented in R survival (https://cran.r-project.org/web/packages/survival/index.html) and survminer (https://cran.r-project.org/web/packages/survminer/index.html) packages.

### Statistical Tests

The two-sample or pairwise mean comparison was conducted using the Mann-Whitney test. The two-sample or pairwise proportion comparison was conducted using Pearson’s chi-squared test. The *p*-value < 0.05, 0.01, and 0.001 were represented by the symbols ∗, ∗∗, and ∗∗∗.

## Results

### The Expression Patterns of N6-Methyladenosine Regulators and Long Noncoding RNAs in Colon Adenocarcinoma

**FIGURE 1:**
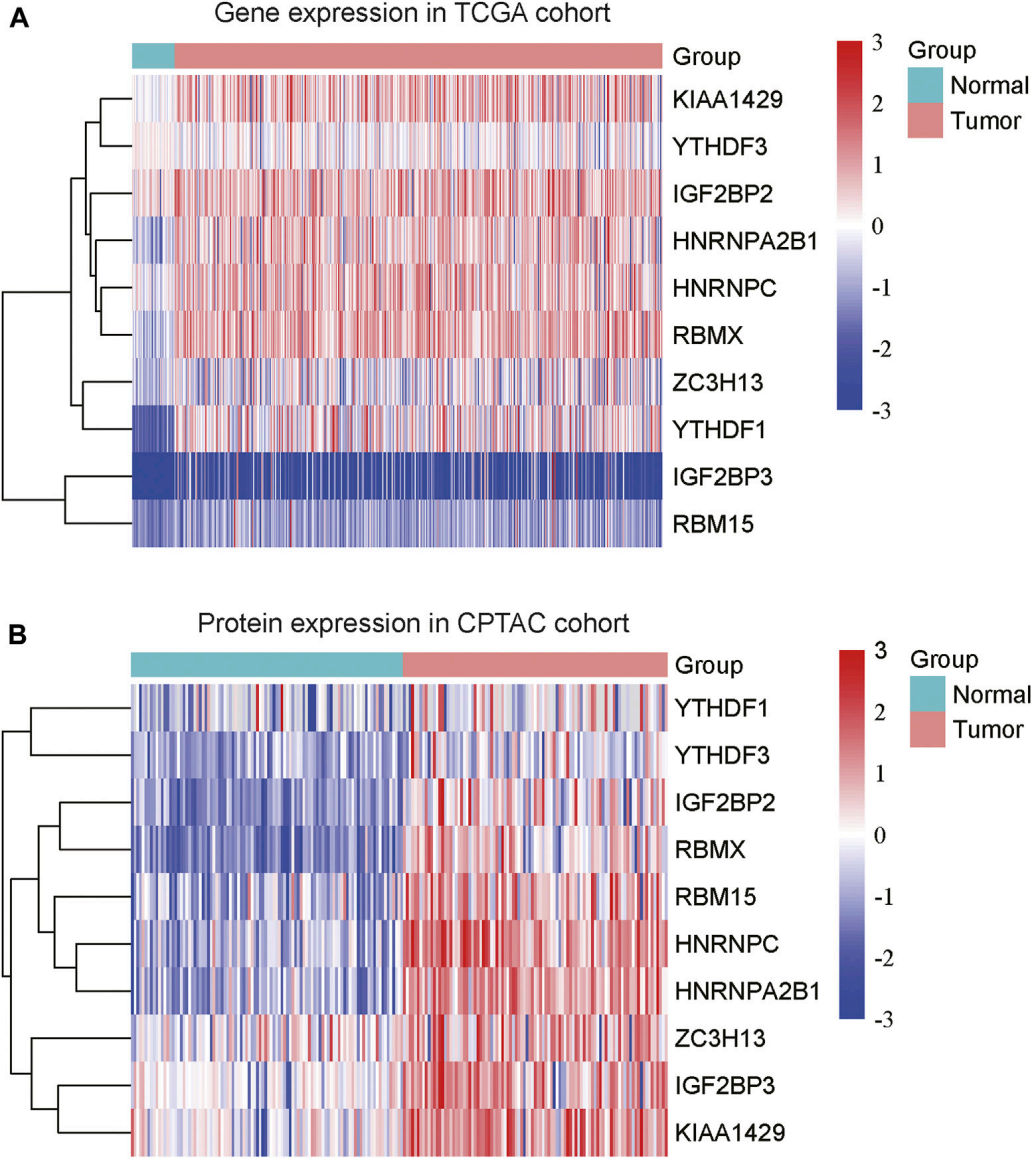
The differentially expressed m6A regulators in COAD. The ten m6A regulators were upregulated in COAD at both mRNA **(A)** and protein **(B)** levels. The expression levels were scaled at −3 to 3.

To explore the expression patterns of m6A regulators and lncRNAs in COAD, we collected the gene expression data from Cancer Genome Atlas (TCGA) and protein expression data from the Clinical Proteomic Tumor Analysis Consortium (CPTAC). Moreover, we also collected a total of 23 m6A regulators from a previous study (Shen et al., 2021), including 8 writers (*METTL3*, *METTL14*, *METTL16*, *RBM15*, *RBM15B*, *WTAP*, *KIAA1429*, and *ZC3H13*), 3 erasers (*ALKBH5*, *ALKBH3*, and *FTO*) and 12 readers (*YTHDC1, YTHDC2, YTHDF1, YTHDF2, YTHDF3, IGF2BP1, IGF2BP2, IGF2BP3, HNRNPA2B1, HNRNPC, RBMX*, and *EIF3A*) (Supplementary Data S1). Specifically, we identified that 10 m6A regulators including 3 writers (*KIAA1429, ZC3H13*, and *RBM15*) and 7 readers (*YTHDF3, IGF2BP2, HNRNPA2B1, HNRNPC, RBMX, YTHDF1,* and *IGF2BP3*) were upregulated in COAD samples at both mRNA and protein levels (Figures 1A,B, adjusted *p*-value < 0.05), as compared with the adjacent normal tissues. Moreover, we also identified 4060 differentially expressed lncRNAs (3,233 upregulated and 827 downregulated) (Supplementary Data S1). The correlation analysis between the DE-lncRNAs and DE-m6A regulators revealed 2,479 m6A-related lncRNAs (Spearman’s correlation >0.3 or < −0.3, Supplementary Data S1).

### Identification of Colon Adenocarcinoma Subtypes by the N6-Methyladenosine-Related Long Noncoding RNAs

**FIGURE 2:**
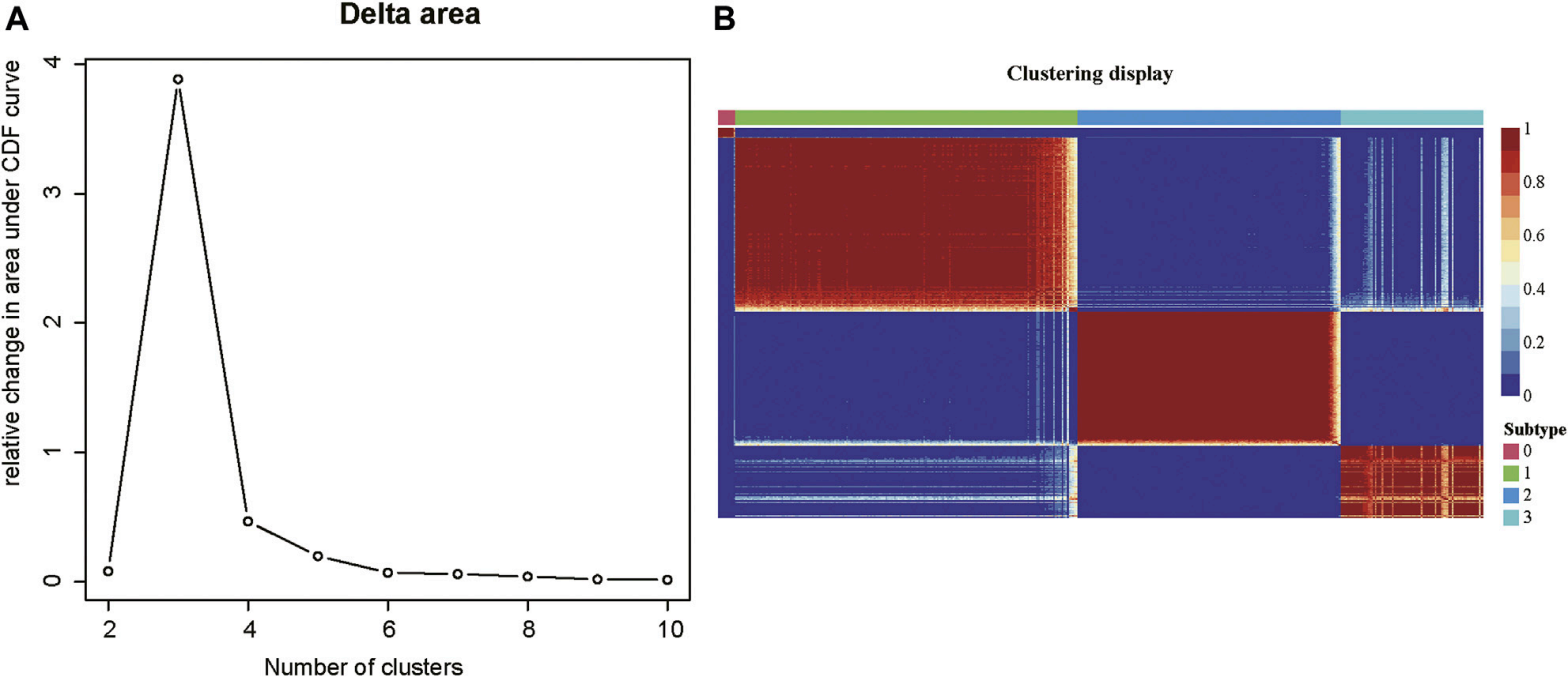
The consensus clustering of the COAD patients by m6A-related lncRNAs. **(A)** The delta areas at cluster numbers from 2 to 10. **(B)** The sample-wise correlation matrix visualized in the heatmap.

As tumor samples often exhibited high heterogeneity, we then investigated whether tumor samples could be divided into multiple subtypes based on those m6A-related lncRNAs. The consensus clustering analysis was conducted on the m6A-related gene expression data, which divided the tumor samples into 2–10 clusters, respectively. The delta area plot indicated that the optimal cluster number was 4, as cluster stability decreased significantly thereafter (Figure 2A). The correlation analysis across the tumor samples revealed that inter-cluster similarity was significantly lower, while the intra-cluster similarity was high. (Figure 2B). Notably, the COAD samples could be divided into four subtypes, each with 11, 200, 154, and 83 samples, respectively. Notably, the subtype 0 contained a small fraction of samples, all of which were formalin-fixed and paraffin-embedded (FFPE). Considering that the FFPE samples had different histological characteristics from the fresh samples, we excluded the subtype 0 in further analysis. Collectively, the m6A-related lncRNAs could clearly stratify the COAD samples into three subtypes.

### Clinical Characteristics of N6-Methyladenosine-Long Noncoding RNAs-Related Subtypes

**FIGURE 3:**
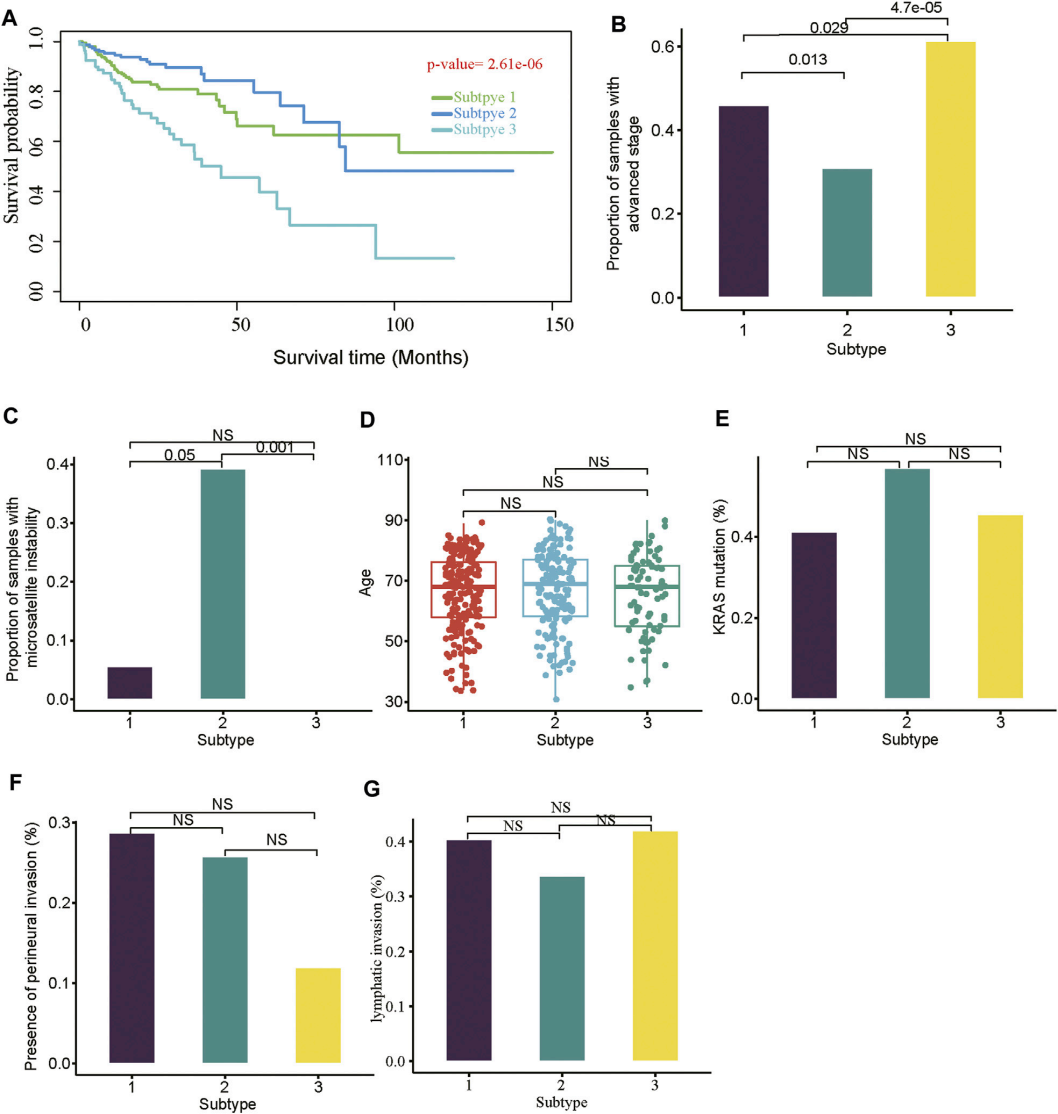
The association of subtypes with the clinical characteristics. **(A)** The differences of overall survival among the three subtypes. The pairwise comparison between the three subtypes identified the differences of **(B)** COAD proportions with advanced stages (TNM stages III and IV), **(C)** proportion of samples with microsatellite instability (MSI), **(D)** age, **(E)** KRAS mutation frequency, **(F)** percentage of perineural invasion, and **(G)** percentage of lymphatic invasion.

With the m6A-lncRNA-related subtypes, we examined its association with the clinical characteristics of COAD patients. Particularly, the subtype-3 had the shortest overall survival when compared with the subtypes 1 and 2 (Figure 3A, log-rank test, *p*-value < 0.05). Consistently, the subtype-3 had a higher proportion of patients with advanced stage than the other two subtypes (Figure 3B). Moreover, we also found that nearly 40% of samples from the subtype 2 were with microsatellite instability (MSI), and such proportion was significantly higher than that observed in the two other subtypes (Figure 3C). In addition, other clinical factors such as patient age, *KRAS* mutation, perineural invasion, and lymphatic invasion were not significantly correlated to this subtyping results (Figures 3D–G). These results indicated that the m6A-lncRNA-related subtypes were prognostically relevant.

### Functional Characterization of N6-Methyladenosine-Long Noncoding RNAs-Related Subtypes

**FIGURE 4:**
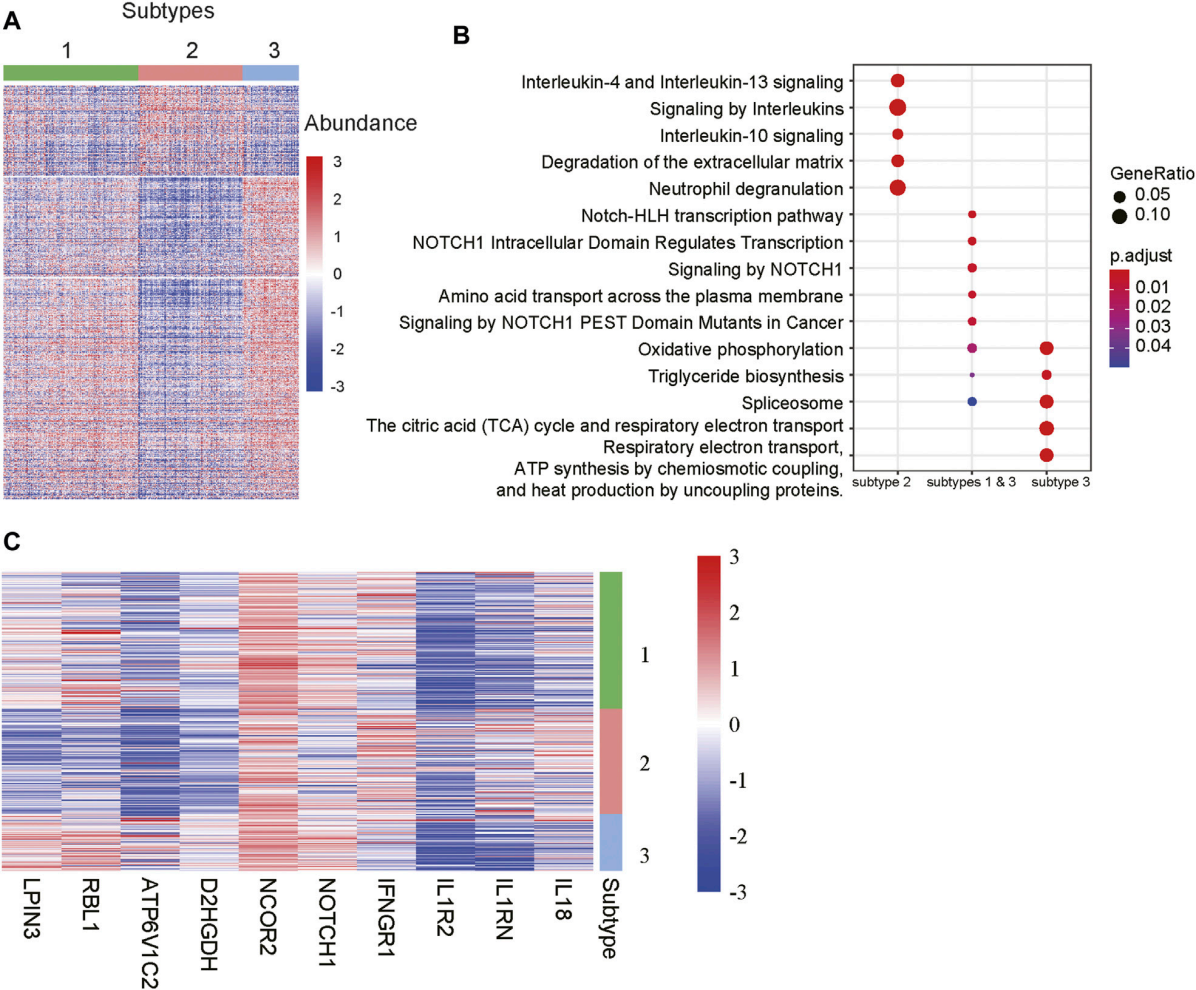
Differentially expressed genes and pathways among the subtypes. **(A)** The gene expression profiles of the differentially expressed genes (DEGs) among the subtypes. The colors on the top represent the subtypes. The red and blue colors in the heatmap indicate high and low expression, respectively. **(B)** The signaling pathways enriched by the DEGs. The x-labels such as subtype 2, subtype 1 & 3, and subtype 3 represent the pathways enriched by the DEGs upregulated in subtypes 2, both 1 and 3, and 3, respectively. **(C)** The representative genes differentially expressed in the subtypes.

To further characterize the functionalities of the m6A-lncRNA-related subtypes, we conducted differential expression analysis and gene set enrichment analysis (GSEA) to identify subtype-specific genes and pathways. Specifically, we identified 4, 210, and 240 genes that were specifically upregulated in subtypes 1, 2, and 3, respectively. It should be noted that those genes were expressed higher in a specified subtype when compared with the other two subtypes Considering that there were few upregulated genes in subtype 1, we also identified 516 genes simultaneously upregulated in subtypes 1 and 3 when compared with subgroup 2. As shown in Figure 4A, those genes exhibited significantly different expression patterns across the subtypes.

The GSEA revealed that the genes upregulated in subtypes 1 and 3 were primarily enriched in NOTCH-related signaling pathways, while those specifically upregulated in subtype 3 were involved in energy metabolism such as oxidative phosphorylation, the citric acid (TCA) cycle and respiratory electron transport, respiratory electron transport, ATP synthesis by chemiosmotic coupling, and heat production by uncoupling proteins (Figure 4B). Notably, the subtype 2 was characterized by the inflammatory response-related pathways like interleukin-4 and interleukin-13 signaling, signaling by interleukins, interleukin-10 signaling, and neutrophil degranulation (Figure 4B). Accordingly, the components involved in NOTCH signaling pathways, such as NOTCH1 and NCOR2, inflammatory factors like *IL18, IL1RN, IL1R2* and *IFNGR1*, and energy metabolism-related genes, such as *D2HGDH, ATP6V1C2, RBL1* and *LPIN3*, were specifically upregulated in the corresponding subtypes (Figure 4C). These results indicated that the m6A-lncRNA-related subtypes had significantly different gene expression patterns and dysfunctional pathways.

### The Subtype-specific N6-Methyladenosine Regulators and Long Noncoding RNAs

**FIGURE 5:**
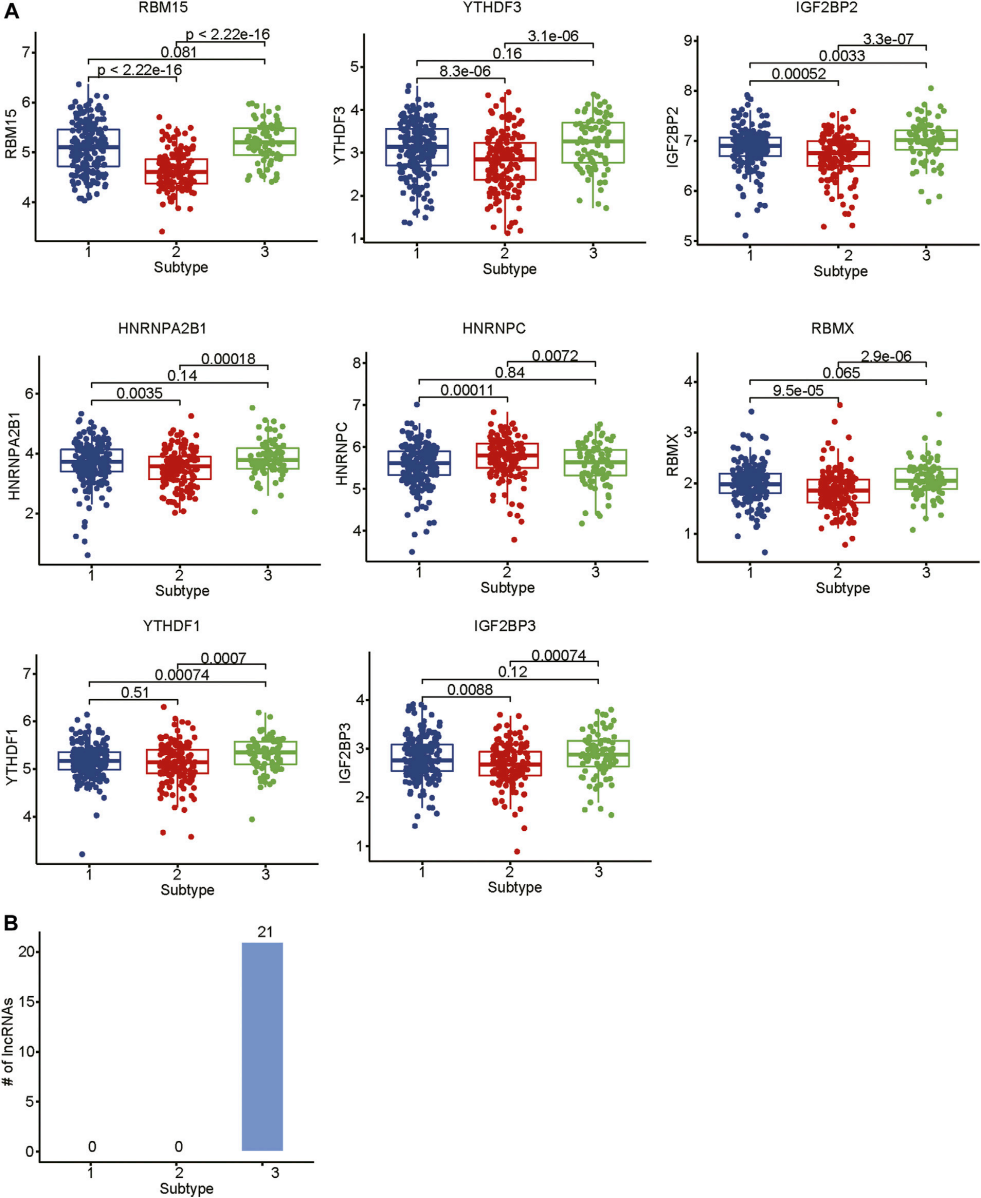
The differential expression levels of m6A regulators and the related lncRNAs among the subtypes. **(A)** The expression levels of m6A regulators in the three subtypes. **(B)** The number of differentially expressed lncRNAs in the three subtypes.

**FIGURE 6:**
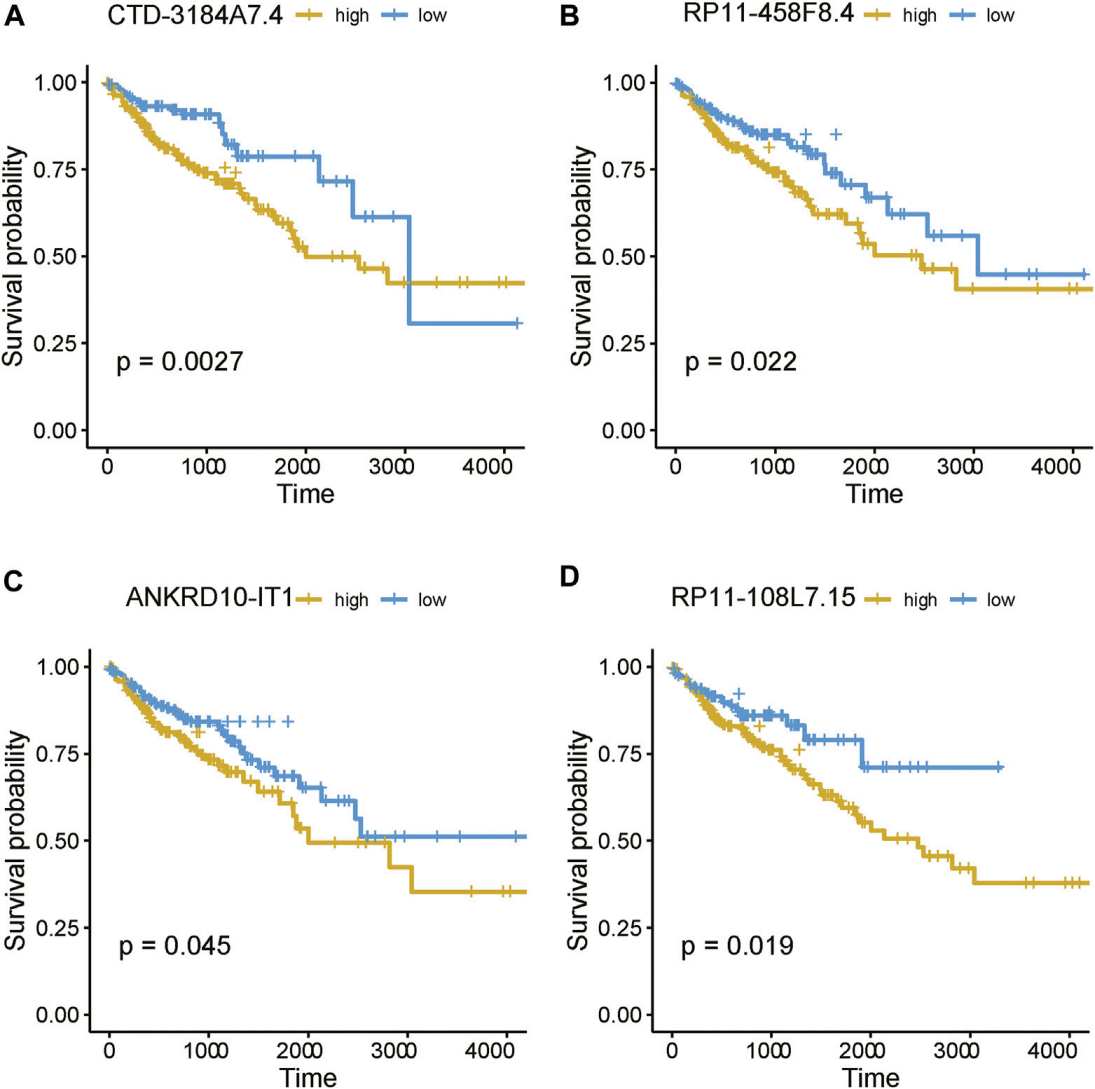
The prognostic values of the four m6A-related lncRNAs. The Kaplan-Meier curves for the samples with high and low expression of **(A)** CTD-3184A7.4, **(B)** RP11-458F8.4, **(C)** ANKRD10-IT1, and **(D)** RP11-108L7.15.

As the m6A regulators were upregulated in COAD, we then investigated whether these genes were upregulated in any specific subtype. Among the ten m6A regulators upregulated in COAD, 8 genes including one writer (*RBM15*) and 7 readers (*YTHDF3, IGF2BP2, HNRNPA2B1, HNRNPC, RBMX, YTHDF1,* and *IGF2BP3*), were specifically upregulated in subtype 3 (Figure 5A, adjusted *p*-value < 0.05). Moreover, we also identified 21 m6A-related lncRNAs as subtype 3 specific, indicating that the m6A readers were closely associated with the biological characteristics of subtype 3 (Figure 5B, adjusted *p*-value < 0.05). More importantly, among the 21 m6A-related lncRNAs in subtype 3, four lncRNAs including *CTD-3184A7.4, RP11-458F8.4, ANKRD10-IT1,* and *RP11-108L7.15* were found to be associated with poor prognosis (Figure 6), suggesting that the m6A regulators and the related lncRNAs were prognostically relevant. In addition, to test whether the m6A proteins could potentially regulate lncRNAs through RNA methylation, we predicted the physical interaction between the lncRNAs and m6a proteins using a deep learning method, LncADeep (Yang et al., 2018b). Notably, RP11-108L7.15 was predicted to interact with HNRNPA2B1 and RBMX, while CTD-3184A7.4 and RP11-458F8.4 might be regulated by YTHDF1 (Table 1), suggesting that the m6A proteins might act as the upstream regulators of the lncRNAs.

**TABLE 1:** The interaction between lncRNAs and m6A proteins by lncADeep.

lncRNA	m6A protein	Spearman’s correlation
RP11-108L7.15	HNRNPA2B1	0.32
RP11-108L7.15	RBMX	0.33
CTD-3184A7.4	YTHDF1	0.63
RP11-458F8.4	YTHDF1	0.44

### Functional Inference of the Four Prognostically Relevant N6-Methyladenosine-Related Long Noncoding RNAs

**FIGURE 7:**
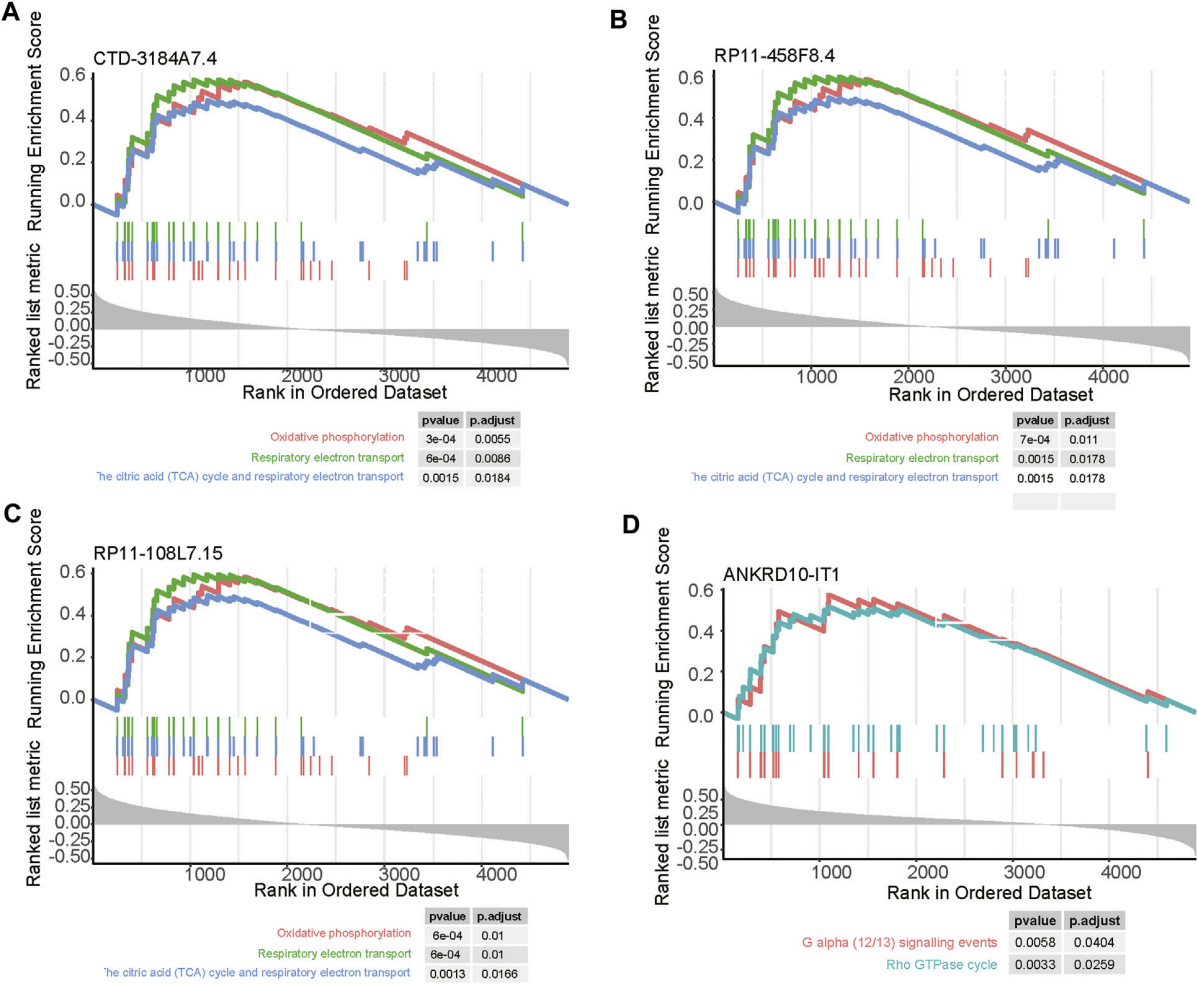
The pathways related to the four m6A-related lncRNAs. The pathways enriched by the positively correlated genes with **(A)** CTD-3184A7.4, **(B)** RP11-458F8.4, **(C)** ANKRD10-IT1, and **(D)** RP11-108L7.15. The vertical lines under the lines represent the genes involved in the pathways. The left and right vertical lines indicated the positively and negatively correlated genes with the lncRNAs.

As the biological functions of the lncRNAs were usually unknown, to shed light on how the four prognostically relevant lncRNAs promoted the tumor progression, we conducted correlation analysis and GSEA. As the four lncRNAs were specifically upregulated in subtype 3, and this subtype was characterized by abnormal activity of energy metabolism, the GSEA further revealed that three out of these lncRNAs, including *CTD-3184A7.4, RP11-458F8.4,* and *RP11-108L7.15* were positively correlated with the energy metabolism-related pathways such as oxidative phosphorylation, respiratory electron transport, the citric acid (TCA) cycle and respiratory electron transport (Figures 7A–C). In contrast, *ANKRD10-IT1* was predicted to be involved in G alpha (12/13) signaling events and Rho GTPase cycle (Figure 7D).

**FIGURE 8:**
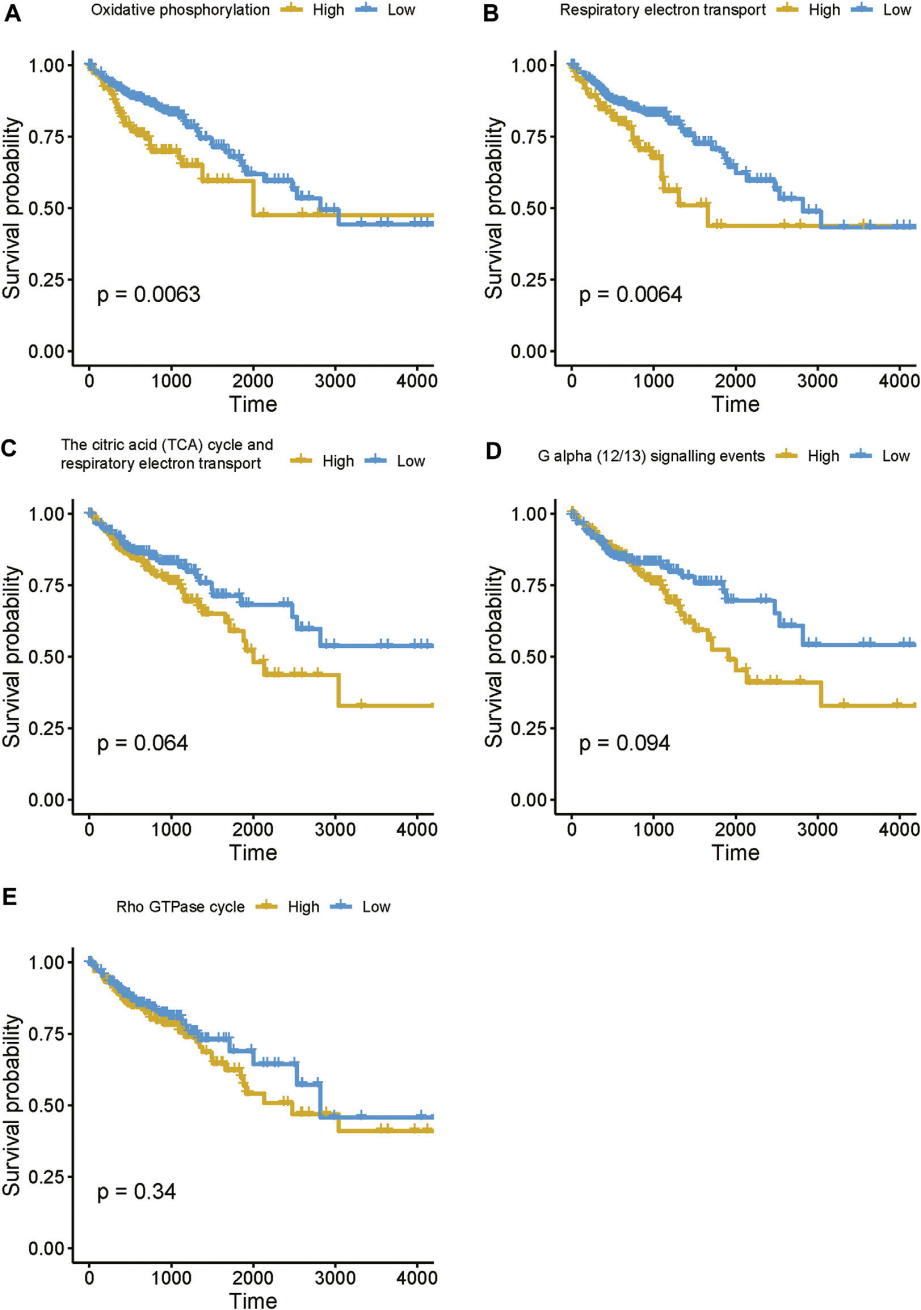
The estimated activities of the lncRNA-related pathways. **(A–E)** The survival curves for the COAD patients with high and low activities of lncRNA-related pathways.

Moreover, to further reveal the expression pattern of genes enriched in these pathways, we estimated the activities of these pathways based on the gene expression data using single-sample gene set enrichment analysis (ssGSEA). The survival analysis revealed that enhanced activities of these pathways were associated with shorter survival time (Figure 8), suggesting that high expression of the lncRNAs associated with these pathways might result in poor prognosis. Taken together, these results suggested that the four prognostically relevant m6A-related lncRNAs might participate in energy metabolism-related pathways, G alpha (12/13) signaling, or Rho GTPase cycle, thereby resulting in unfavorable outcomes.

## Discussion

N6-methyladenosine (m6A) is one of the most prevalent RNA modifications in mRNAs and non-coding RNAs that regulates splicing, translation, stability of protein-coding RNAs, and epigenetic effects of certain non-coding RNA (He et al., 2019). However, the functional effects of m6A-related lncRNAs in colon adenocarcinoma (COAD) has not been fully appreciated.

In this study, we identified 10 m6A regulators that were upregulated in COAD samples at both mRNA and protein levels, and 4,060 differentially expressed lncRNAs in COAD. The correlation analysis between the DE-lncRNAs and DE-m6A regulators identified 2,479 m6A-related lncRNAs (Spearman’s correlation >0.3 or < −0.3). As the lncRNAs were associated with cancer subtypes (Cedro-Tanda et al., 2020; Arnes et al., 2019), the m6A-related lncRNAs could also clearly stratify the COAD samples into three subtypes. We found that nearly 40% of samples in the subtype 2 had microsatellite instability (MSI), which was significantly higher than in the two other subtypes. In accordance with this finding, the inflammatory response-related pathways were highly activated in this subtype. It is well known that high inflammation is closely associated with more neo-antigens due to MSI (Lin et al., 2020). Moreover, the comparative analysis of the clinical characteristics between the subtypes revealed that subtype-3 had a shorter overall survival and a higher proportion of patients with advanced stage than subtypes 1 and 2 (*p*-value < 0.05). The pathway analysis suggested that the energy metabolism-related pathways might be aberrantly activated in this subtype. Notably, D-2hydroxyglutarate dehydrogenase (D2HGDH), which was upregulated in subtype 3, was observed to drive progression to colorectal cancer during colitis (Han et al., 2018). Accumulating evidence has demonstrated that RNA modification exerts extensive effects on the cancer metabolic network (Han et al., 2020). Consistently, we observed that most of the m6A readers (*YTHDF3, IGF2BP2, HNRNPA2B1, HNRNPC, RBMX, YTHDF1,* and *IGF2BP3*) and 21 m6A-related lncRNAs were upregulated in subtype 3, suggesting that the m6A readers and the m6A-related lncRNAs might be associated with metabolic reprogramming and unfavorable outcomes in COAD. The m6A readers have been reported to have prognostic values in colorectal cancer (Ji et al., 2020; Liu et al., 2019). Among those m6A-related lncRNAs in subtype 3, four were predicted as prognostically relevant. Functional inference of these lncRNAs suggested that *CTD-3184A7.4, RP11-458F8.4,* and *RP11-108L7.15* were positively correlated with the energy metabolism-related pathways such as oxidative phosphorylation, respiratory electron transport, the citric acid (TCA) cycle and respiratory electron transport, further suggesting that these lncRNAs might be involved in energy metabolism-related pathways. The RNA-protein interaction prediction revealed that RP11-108L7.15 might interact with HNRNPA2B1 and RBMX, while CTD-3184A7.4 and RP11-458F8.4 might be regulated by YTHDF1 (Table 1), suggesting that the m6A proteins might act as the upstream regulators of the lncRNAs. Remarkably, *CTD-3184A7.4*, also termed as *MHENCR*, and RP11-108L7.15 have been reported to promote melanoma progression via PI3K-Akt signaling (Chen et al., 2017), and cell proliferation, migration and invasion in glioblastoma (Shi et al., 2017), further suggesting that these lncRNAs might play functional roles in COAD progression. Meanwhile, the m6A proteins have been widely reported to regulate lncRNAs via RNA methylation in several cancers (Dai et al., 2018; Zhang et al., 2019; Guo et al., 2020).

In addition, the present study has some limitations such as the lack of experimental validation for the key regulators and clinical validation for the association between those regulators and clinical characteristics. Moreover, the regulatory relationship between the lncRNA and m6A regulator is difficult to be inferred by the correlation analysis solely, and more experimental data is urgently needed to demonstrate their upstream and downstream mechanisms. In summary, we conducted a systematic data analysis to identify the key m6A regulators and m6A-related lncRNAs, and evaluated their clinical and functional importance in COAD, which may provide important evidences for further m6A-related researches.

## Data Availability

The original contributions presented in the study are included in the article/Supplementary Material, further inquiries can be directed to the corresponding author.
